# Barriers and facilitators to implementing childcare in long-term care homes: A scoping review protocol and consultative exercise

**DOI:** 10.1371/journal.pone.0350032

**Published:** 2026-05-27

**Authors:** Kristina M. Kokorelias, Annie Robitaille

**Affiliations:** 1 Department of Geriatric Medicine, Sinai Health System and University Health Network, Toronto, Ontario, Canada; 2 Department of Occupational Science & Occupational Therapy, Temerty Faculty of Medicine, University of Toronto, Toronto, Ontario, Canada; 3 Rehabilitation Sciences Institute, Temerty Faculty of Medicine, University of Toronto, Toronto, Ontario, Canada; 4 KITE- University Health Network, Toronto, Ontario, Canada; 5 Centre of Excellence in Frailty-Informed Care, Perley Health, Ottawa, Ontario, Canada; 6 Faculty of Health Sciences, University of Ottawa, Ottawa, Ontario, Canada‌‌; SKYDA Health Nigeria, NIGERIA

## Abstract

**Background:**

Long-term care (LTC) homes face persistent workforce recruitment and retention challenges, particularly among staff balancing professional responsibilities with childcare needs. Integrating childcare services within LTC homes has the potential to improve staff well-being, workforce stability, and resident experiences through intergenerational engagement. Despite this potential, the implementation literature remains fragmented, and no systematic synthesis of barriers, facilitators, or contextual determinants exists.

**Objective:**

To systematically map the literature on barriers and facilitators to implementing childcare services within LTC homes and identify gaps to inform research, policy, and practice.

**Methods:**

We will conduct a scoping review following the Arksey and O’Malley framework, enhanced by Levac et al., and report findings according to PRISMA-P and PRISMA-ScR guidelines. Literature searches will be conducted in MEDLINE, CINAHL, Embase, Scopus, and PsycINFO, supplemented by grey literature searches. Eligible studies include qualitative, quantitative, or mixed-methods research, program evaluations, and policy reports examining implementation of childcare services within or linked to LTC homes. Two reviewers will independently screen studies, extract data using a standardized form, and resolve discrepancies through discussion or a third reviewer. Extraction will capture study characteristics, childcare model details, reported barriers and facilitators, and outcomes. Findings will be synthesized narratively and organized thematically using the Consolidated Framework for Implementation Research (CFIR 2.0). Stakeholder engagement with LTC and early childhood centre staff will guide interpretation and knowledge translation.

**Expected outcomes:**

The review will identify key determinants of successful implementation, highlight gaps in the evidence, and provide actionable insights for LTC administrators, early childhood partners, and policymakers seeking to develop sustainable, equitable co-located childcare programs that benefit residents, children, and staff.

## Introduction

Integrating childcare into long-term care (LTC) homes support the creation of age-friendly, socially connected environments for older adults, while also expanding access to reliable childcare for working families and care staff [1,2]. Intergenerational programming, broadly defined as structured activities or shared spaces that bring children and older adults into regular, purposeful contact, has a long history within residential care settings [3] and has been associated with social, emotional, and cognitive benefits for participants of both generations [4,5]. Empirical reviews and program reports point to improvements in stimulation, mood, social engagement, and feelings of purpose among older adults, alongside social and developmental advantages for children who gain exposure to older role models and diverse social relationships [4,6,7].

Despite these promising effects, relatively few LTC homes actually embed childcare services on site or operate truly shared-site intergenerational care centers [8,9]. Where they do exist, models can include fully co-located early learning centres that share programming and physical space with a nursing home [8,10]. Examples of intentional co-location and integrated programming have been documented in municipalities that reconfigured site design and operations to encourage routine interactions, but these examples remain limited in number and in the published implementation science literature [11,12].

Embedding a childcare facility within LTC homes presents potential benefits that reach multiple interest groups. For residents, consistent contact with children can increase daily engagement, reduce loneliness, and provide meaningful roles that support dignity and identity [13,14]. For children, structured interactions with older adults support social skills, intergenerational understanding, and exposure to diverse life experiences [4,15]. For LTC staff and organizations, on-site childcare may improve recruitment and retention by reducing childcare-related barriers to workforce participation and by strengthening ties with families and the surrounding community [16]. For policy makers and planners, shared-site models offer a strategy to make better use of community assets and to address parallel shortages in eldercare and childcare infrastructure [8]. Yet these potential benefits do not automatically translate into sustainable programs. Practical, regulatory, cultural, and financial barriers can limit feasibility, and programs that appear promising in one context may struggle to scale or spread without careful attention to implementation factors [2].

Although descriptive accounts and small-scale evaluations document outcomes and participant experiences, the implementation evidence base for childcare embedded in LTC homes is not well synthesized. Key questions remain unanswered such as what organizational features enable or block co-location, how do regulatory and funding systems shape possibilities, what adaptations are needed to accommodate vulnerable groups such as people living with dementia, and how do staff, families, and community partners experience and sustain these initiatives over time? Answering these questions requires an approach that systematically maps implementation determinants across multiple levels and that identifies where the literature is lacking to inform other organizations looking to implement these models.

The updated Consolidated Framework for Implementation Research (CFIR 2.0) can be a guiding conceptual lens for understanding barriers and facilitators to implementation of complex interventions, such as co-locating childcare within LTC homes [17]. CFIR 2.0 provides a structured taxonomy of determinants organized into interrelated domains including the innovation or intervention characteristics, the outer setting, the inner setting, the individuals involved, and the implementation process [17]. The 2022 update from the original CFIR explicitly adds attention to innovation recipients and to equity-related determinants, to make the CFIR 2.0 well suited for both prospective and retrospective analyses of implementation efforts in diverse settings [17].

There are several published works investigating intergenerational programming and shared-site models that bring older adults and children together in structured or informal activities. To our knowledge, there is no published scoping review that specifically examines the barriers and facilitators to implementing childcare within long-term care homes. A focused synthesis of implementation barriers and facilitators is therefore needed to inform the design and scale-up of integrated childcare models within LTC homes. This review aims to map the existing literature on how childcare programs are developed, integrated, and sustained in long-term care settings, with particular attention to the factors that support or hinder implementation. The findings may inform future planning, policy decisions, and organizational strategies for long-term care leaders and childhood education partners who are considering co-located or shared-site childcare models.

### Theoretical framework

This review is guided by the CFIR 2.0. CFIR 2.0 organizes implementation determinants across five interconnected domains: the characteristics of the innovation, the outer setting, the inner setting, the individuals involved, and the implementation process [17]. The updated version places greater emphasis on recipients of an innovation, equity considerations, and the dynamic interactions across levels that shape how programs are adopted, integrated, or sustained [17].

CFIR 2.0 is particularly well suited to the context of embedding childcare within long-term care homes because this is a multi-level innovation that requires alignment among regulatory bodies, organizational cultures, physical environments, staff beliefs, family expectations, and resource structures (often across multiple agencies) [17]. Childcare co-located within LTC also represents a shift in how care spaces are traditionally conceived, that means implementation is influenced not only by operational logistics but also by values, norms, and interprofessional relationships [10]. By using CFIR 2.0, this review can capture determinants that may otherwise be overlooked, such as how staff and family perceptions shape acceptability, and how leadership engagement or funding mechanisms support or hinder uptake. Additionally, applying CFIR 2.0 to this scoping review provides a structured way to extract and categorize the disparate evidence that exists, allowing comparison across studies that vary in context, design, and maturity of implementation [10].

Review questions

1 What is known about the implementation of childcare within long-term care homes and how does implementation influence staff and resident outcomes?
  a What barriers to implementing childcare in long-term care homes are reported in the literature?
  b What facilitators to implementing childcare in long-term care homes are reported in the literature?
2 What gaps remain in the evidence base regarding the planning, implementation, and sustainability of childcare models in long-term care settings?

## Methods

A scoping review was selected to capture the full range of evidence on childcare implementation in long-term care homes. This approach supports a broad examination of what has been studied, the types of evidence available, and how different study designs have approached this topic [18]. We will follow the Joanna Briggs Institute scoping review methodology [19]. The protocol is reported in line with Preferred Reporting Items for Systematic review and Meta-Analysis Protocols (PRISMA-P) [20], and the final review will be reported using The PRISMA extension for scoping reviews (PRISMA-ScR) framework [21].

### Protocol and registration

The protocol was registered on Open Science Framework on January 24, 2026 (osf.io/ywtvz)

### Community engagement strategy

An integrated knowledge translation (iKT) approach will guide this scoping review, involving co-production of research with individuals who have lived experience in early childhood and long-term care settings [22]. Guided by the Canadian Institutes of Health Research’s Patient Engagement Framework and the principles of mutual respect, inclusiveness, and co-building, the review will engage staff from one early childhood care centre and one LTC facility to bring practical perspectives on implementing childcare in LTC [23].

Partners will contribute to the development of the data extraction tool, advise on study relevance, review preliminary results, and provide feedback on interpretation and dissemination. Roles and responsibilities will be clearly defined, and partners will have flexibility in how they participate. Meetings will be held virtually and scheduled to accommodate participants, with opportunities for synchronous and asynchronous contributions. Partners will be acknowledged as co-authors (if they wish) on the resulting publication to reflect their essential contributions.

Ethical oversight is not required for staff partners, as they will participate as team members rather than study subjects. Nevertheless, the review team will ensure ethical engagement, including confidentiality, accessible communication, and shared decision-making. Training and ongoing support will be provided to enable meaningful participation.

### Inclusion criteria

Participants: Studies will be included if they involve staff, administrators, or other stakeholders working in long-term care (LTC) homes, including healthcare workers, support staff, and management. Studies that report on children, families and/or LTC home residents who access childcare services located within or affiliated with LTC homes will also be eligible.

Concept: This review will include English or French-language studies or evaluations that examine the implementation of childcare services or programs within LTC homes. Eligible studies will focus on barriers, facilitators, strategies, or outcomes related to implementing childcare for staff or residents’ families. All types of evidence, including qualitative, quantitative, mixed-methods studies, program evaluations, and policy reports, will be considered.

Context: Studies must be conducted in long-term care homes or nursing homes, including residential aged care settings. Childcare services must be provided on-site, integrated into the facility, or directly linked to supporting LTC staff or families. No restrictions will be applied regarding country or healthcare system.

#### Sources.

This review will include studies using any empirical research method and will incorporate both published and unpublished sources that investigate the implementation of childcare services within LTC homes. Literature from any country and published in English or French will be considered to capture a range of cultural and healthcare contexts. No restrictions will be placed on publication year. Review articles will not be included, however reference lists of all relevant reviews and studies will be examined to identify additional sources. Where conference abstracts are subsequently published as full peer-reviewed articles, these will also be included.

Grey literature will be eligible if it provides empirical or programmatic evidence on the implementation of childcare in LTC settings, including reports, theses, policy documents, and organizational evaluations. Only grey literature with sufficient methodological detail to extract relevant implementation data will be included.

### Search strategy

An academic research librarian will support the development of the electronic database search strategy. An initial search will be conducted in Medline (Ovid) using MeSH terms and text words for the following three concepts: long-term care, childcare, and implementation (see Supplemental Material 1). The review team will identify additional relevant terms and synonyms to include. The initial Medline search will be peer-reviewed using the Peer Review of Electronic Search Strategies (PRESS) guideline [24]. The final search strategy will then be translated across the following databases: CINAHL (EBSCOhost), Embase (Ovid), Scopus, and PsycINFO (ProQuest). No restrictions will be applied to publication year. Reference lists of included studies and relevant grey literature will be screened to identify additional sources. Grey literature, including government reports, policy documents, organizational websites, and conference proceedings, will be searched using targeted web searches (Google), relevant organizational repositories, and consultation with experts in LTC and early childhood programs. Searches will be limited to English and French.

### Study/Source of evidence selection

Citations from all searches will be uploaded into Covidence. Covidence will remove duplicates [25]. Two reviewers will screen an initial random sample of 100 titles and abstracts to establish inter-rater reliability, aiming for a Cohen’s Kappa of 0.80 to indicate substantial agreement [26]. After agreement is reached, two reviewers will independently screen all remaining titles and abstracts against the inclusion criteria. Any citations marked as uncertain will move forward to full-text review. Full texts will then be retrieved in Covidence and screened independently by two reviewers using the same criteria, with reasons for exclusion recorded. Any disagreements at either stage will be resolved through discussion with a third reviewer when needed.

### Data extraction

Data from included full texts will be systematically charted by two reviewers in Covidence using a data extraction template informed by the JBI guidance [27], the CFIR 2.0 [17] and refined by the review team. Extracted information will include author(s), year of publication, country, study purpose, study design, setting, population characteristics, and details of the childcare model or intergenerational program described, as well as their outcome. To support the implementation focus of this review, we will extract all reported barriers, facilitators, and contextual factors relevant to introducing or sustaining childcare within long-term care homes. These determinants will be mapped to CFIR 2.0 domains and constructs, including characteristics of the innovation, outer setting influences, inner setting features such as leadership or culture, characteristics of individuals involved, and processes used to plan, implement, or adapt the childcare model [17]. Additional information on implementation outcomes, reported impacts, and any recommendations or lessons learned will also be documented [17]. Two reviewers will independently extract data, and discrepancies will be resolved through consultation with a third reviewer.

### Data analysis and presentation

The primary author (KMK) will first collate, summarize, and report the results, with feedback from the research team. Quantitative data will be organized into tables and described using basic statistics such as frequencies and percentages, with narrative explanation where helpful. Qualitative findings will be synthesized using a conventional content analysis approach to generate a descriptive summary [28]. The search has been translated across all databases and preliminary title and abstract screening will begin in June 2026, data extraction by September 2026, and synthesis by January 2027.

### Community engagement strategy and ethical considerations

An integrated knowledge translation (iKT) approach will guide this scoping review, involving collaboration with knowledge users to enhance the relevance and applicability of findings. Knowledge users include staff from one long-term care facility and one early childhood care centre. These individuals will be engaged as members of the research team in an advisory capacity rather than as research participants. Their role will be to support interpretation of findings, contribute to refinement of implementation considerations, and provide input into knowledge translation outputs. They will also provide feedback on the relevance and clarity of extracted findings and assist in identifying practical considerations for implementation in real-world settings. Engagement activities will include structured virtual meetings and asynchronous input opportunities. Participants will be invited to contribute to the development of the data extraction framework, review emerging synthesis findings, and provide contextual feedback on preliminary interpretations. Participation will be flexible, and individuals may contribute according to their availability and preferred mode of engagement. No data will be collected from these knowledge users for research purposes, and their contributions will not be treated as study data.

Ethics approval was not required for this scoping review protocol and is waived for the full study. The consultative exercise involves engagement with knowledge users and stakeholders in an advisory capacity to support interpretation of findings and refinement of knowledge translation outputs. These consultations do not involve collection of personal information or any form of research participation as defined by institutional research ethics guidelines. Engagement is limited to advisory input to support interpretation and dissemination of findings. Ethical engagement principles, including confidentiality in group discussions, respectful collaboration, and accessible communication, will be upheld throughout the project.

## Discussion

This paper outlines the protocol for a forthcoming scoping review (expected by end of 2026). This scoping review will provide a comprehensive synthesis of the literature on implementing childcare within LTC homes, an area that remains underexplored despite the amount literature on the potential benefits of intergenerational programming. Embedding childcare directly within LTC homes has the potential to enhance social engagement and purpose among residents, support children’s development through meaningful interactions with older adults, and improve workforce participation by reducing childcare barriers for staff [29,30]. However, translating these benefits into sustainable programs is contingent on understanding the multi-level factors that shape implementation.

By mapping barriers and facilitators using the CFIR 2.0 framework [17], this review will highlight key determinants across the innovation, outer setting, inner setting, individuals, and implementation process domains. This structured approach will allow identification of operational, organizational, cultural, and policy-related influences that may support or hinder co-located childcare programs. Attention to equity and recipient perspectives, emphasized in CFIR 2.0 [17], will further support understanding of how diverse populations, including residents with dementia, experience these programs.

The review will also identify gaps in the literature including any areas where there is scarce reporting of sustainability outcomes, and inconsistent documentation of adaptations for different LTC contexts. Taken together, findings can inform practical guidance for LTC administrators, early childhood partners, and policy makers considering integrated childcare models through supporting evidence-informed planning for co-location, resource allocation, staff training, and intersectoral collaboration.

Finally, engaging staff from both a LTC facility and an early childhood centre in this review ensures that findings are grounded in practical realities, enhancing the relevance and applicability of results. Insights gained through this integrated knowledge translation approach can guide both future research and real-world implementation, ultimately contributing to age-friendly, socially connected, and family-supportive LTC environments.

However, this review will proceed with some limitations. First, it is restricted to studies published in English or French, that may exclude relevant evidence from other linguistic and cultural contexts. Secondly, although the use if the CFIR 2.0 provides a structured framework for mapping implementation determinants, the review relies on how well studies report implementation processes; underreporting or inconsistent terminology may result in incomplete mapping of barriers and facilitators. Finally, engagement is limited to staff from one early childhood care centre and one LTC facility, that may not reflect the full range of perspectives across different organizational, geographic, or regulatory contexts.

## Conclusion

Integrating childcare co-located within LTC homes offers opportunities to enhance intergenerational connections, support residents’ well-being, and address workforce childcare needs. This paper presents the protocol for a scoping review that will map the existing evidence on implementation barriers and facilitators, highlighting critical factors across organizational, regulatory, and interpersonal domains. By identifying gaps and lessons learned, the review aims to inform LTC administrators, early childhood partners, and policy makers seeking to develop sustainable, co-located childcare programs that benefit both older adults and children.

## Supporting information

S1 File Prisma-SCr. (DOCX)

S2 File Prisma-P. (DOCX)

S3 File Search Strategy. (DOCX)
