# Supplementary material for: Barriers and facilitators to implementing childcare in long-term care homes: A scoping review protocol and consultative exercise
Source: PLoS One. 2026 May 27;21(5):e0350032. doi: 10.1371/journal.pone.0350032 (PMC13215540; doi:10.1371/journal.pone.0350032)
Supplement: S3 File — (DOCX) [file pone.0350032.s003.docx]

**Ovid MEDLINE(R) ALL <1946 to February 03, 2026>**

**1 Long-Term Care/ or Homes for the Aged/ or Housing for the Elderly/ 45418**

2 ((long adj1 term adj1 care) or "long term care").ti,ab,kf,kw. 31895

3 ltc.ti. 408

4 ((home* or house* or housing* or residen* or centre* or center*) adj1 (aged or "old age" or senior* or elder* or retire* or "contin* care*" or "life care*")).ti,ab,kf,kw. 17822

5 (care adj1 (aged or "old age" or senior* or elder* or retire*)).ti,ab,kf,kw. 9377

6 ((old or older or elder* or senior*) adj1 (adult* or people* or person*)).ti,ab,kf,kw. 264885

7 or/1-6 331461

**8 Child Care/ or Child Day Care Centers/ or Child/ or Child, Preschool/ or Infant/ 2597539**

9 (daycare* or "day care*" or nurser* or preschool* or "pre school*").ti,ab,kf,kw. 70957

10 ((child* or infant* or baby or babies or toddler*) adj2 (care* or educat* or program* or centre* or center* or support*)).ti,ab,kf,kw. 100756

11 (child* or toddler* or "school age").ti,ab,kf,kw. 1900774

12 or/8-11 3217394

**13 Intergenerational Relations/ 4949**

14 (intergeneration* or "inter generation*" or "cross generation*" or multigeneration* or "multi generation*" or transgeneration*).ti,ab,kf,kw. 23176

15 (generation* adj2 (gap or gaps or young* or old or older or relation* or program* or support* or visit* or facilitat*)).ti,ab,kf,kw. 10565

16 or/13-15 35783

*17 7 and 12 and 16 805*

*18 limit 17 to (guideline or practice guideline or meta analysis or "review" or "systematic review" or "scoping review") 56*

*19 17 not 18 749*
